# Clip-assisted anchoring method to facilitate submucosal tunnel entry in peroral endoscopic myotomy for severe fibrosis

**DOI:** 10.1055/a-2734-0493

**Published:** 2025-11-18

**Authors:** Kazuki Yamamoto, Yohei Nishikawa, Kohei Shigeta, Kei Ushikubo, Ippei Tanaka, Satoshi Abiko, Haruhiro Inoue

**Affiliations:** 1 378609 Digestive Diseases Center, Showa Medical University Koto Toyosu Hospital, Koto, Japan

Peroral endoscopic myotomy (POEM) is an established treatment for esophageal achalasia, with technical success rates of 90% to 100% 1 2 3 . However, severe submucosal fibrosis (SMF) can make submucosal tunnel entry challenging and may lead to procedure failure 4 . Therefore, we developed the Clip-assisted Anchoring Method to secure a stable foothold for the endoscope, improving tunnel entry in difficult SMF cases.

**Fig. 1:**
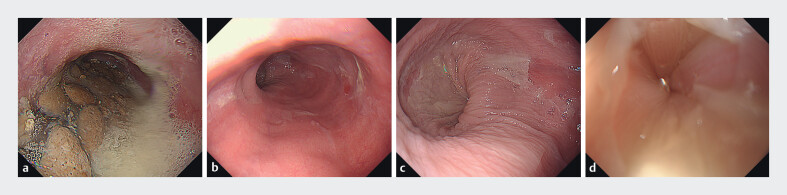
**a** Achalasia patient before peroral endoscopic myotomy (POEM) showing massive food residue in the esophagus. **b** Esophageal lumen after clearance of the food residue prior to POEM. **c** Inflamed esophageal mucosa with epithelial damage and peeling. **d** Rosette-like appearance of the lower esophagus.

**Fig. 2:**
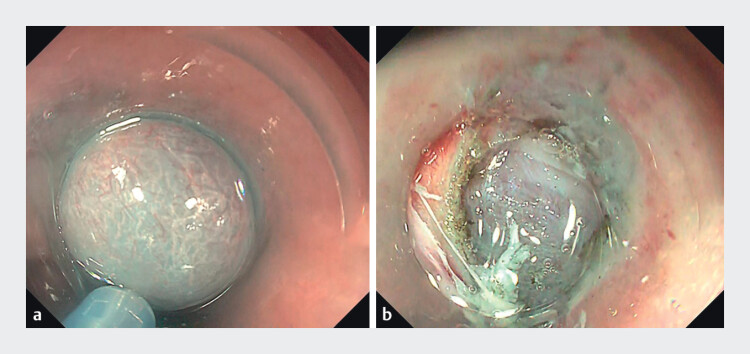
**a** Submucosal injection showing limited lifting effect due to severe fibrosis. **b** Severe fibrosis caused the scope to slip, making tunnel entry difficult.

**Fig. 3:**
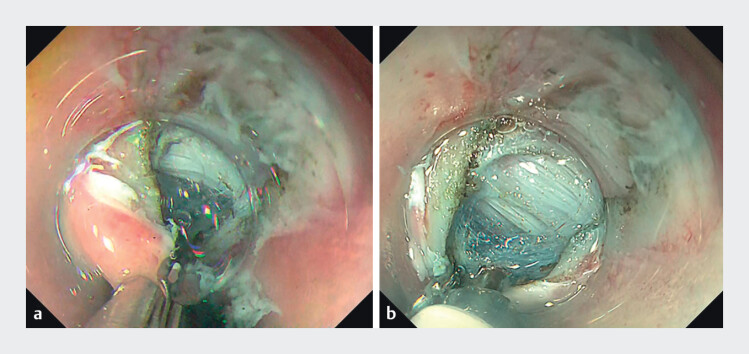
**a** single endoclip (HX-610–090; Olympus) was applied to the distal edge of the incision, creating a stable anchoring point. **b** Clip-assisted Anchoring Method improved stability and visualization and enabled smooth, controlled entry.

A 27-year-old woman with type I achalasia ( Fig. 1 **a-d** ) underwent POEM using a therapeutic endoscope (GIF-H290; Olympus) with a Triangle Tip Knife J (TTJ; Olympus) and an electrosurgical unit (VIO3; ERBE, Endocut I: 1–3-3). A submucosal injection and mucosal incision were made at the 2 o’clock position; however, severe SMF limited the lifting effect, causing the scope to slip and making tunnel entry challenging ( Fig. 2 **a,b** ). Applying a single endoclip (HX-610–090; Olympus) to the distal edge of the incision created a stable anchoring point, markedly improving stability, visualization, and allowing smooth, controlled entry ( Fig. 3 **a,b** ).

**Fig. 4:**
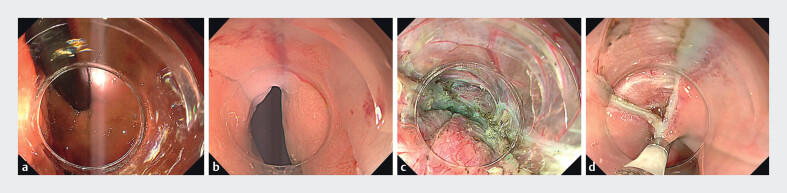
**a** Submucosal dissection proceeded along the lesser curvature. The double-scope technique confirmed tunnel length and orientation in a retroflexed view. **b** Forward view using the double-scope technique showing the submucosal tunnel along the lesser curvature of the stomach. **c** Myotomy was completed at the distal end of the submucosal tunnel. **d** The entry site was completely closed with endoclips (HX-610–090; Olympus).

Once entry was achieved, submucosal dissection proceeded along the lesser curvature. The double-scope technique 5 was used to confirm tunnel length and orientation in both retroflexed and forward views. Myotomy was completed at the distal tunnel, and the entry site was fully closed with endoclips ( Fig. 4 **a-d** and Video 1 ).

Clip-assisted Anchoring Method for Tunnel Entry in POEM.Video 1

**Fig. 5:**
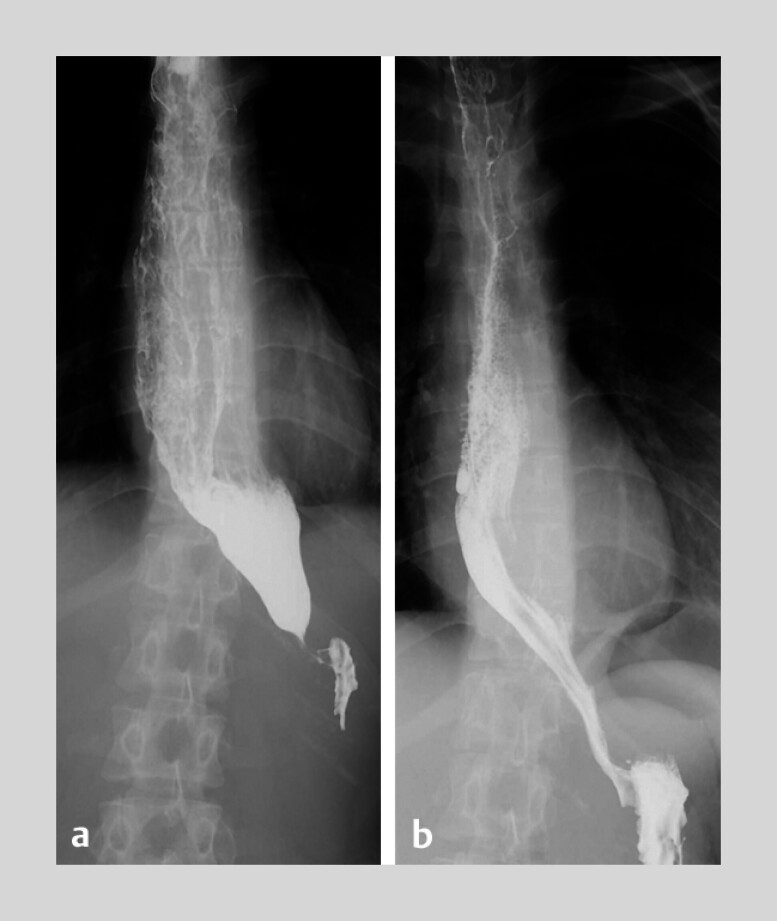
**a** Barium swallow before peroral endoscopic myotomy (POEM). **b** Barium swallow after POEM.

The patient had an uneventful recovery. A postoperative barium swallow showed improved esophageal emptying at the lower esophageal sphincter ( Fig. 5 **a,b** ). Clear liquids were started on day 1, followed by gradual diet advancement. Symptoms resolved and she was discharged on day 4.

This case demonstrates that the Clip-assisted Anchoring Method, by placing a clip at the distal edge of the submucosal incision, provides a stable anchor for the endoscope and enables successful tunnel entry in POEM even with severe fibrosis.
